# The effect of breastfeeding on spontan resolution of monosymptomatic enuresis

**DOI:** 10.1590/S1677-5538.IBJU.2015.0485

**Published:** 2016

**Authors:** Eyup Burak Sancak, Ural Oguz, Aykut Aykac, Erhan Demirelli, Omer Faruk Bozkurt, Sertac Cimen

**Affiliations:** 1 Department of Urology, Canakkale Onsekiz Mart University, Faculty of Medicine, Canakkale, Turkey;; 2 Department of Urology, Giresun University, School of Medicine, Giresun, Turkey;; 3 Department of Urology, Bursa Orhangazi State Hospital, Bursa, Turkey;; 4 Department of Urology, Kecioren Training and Research Hospital, Ankara, Turkey;; 5 Diskapi Yildirim Beyazit Training and Research Hospital, Department of Urology, Ankara, Turkey

**Keywords:** Breast Feeding, Breast Milk Expression, Enuresis

## Abstract

**Purpose:**

The aim of this study was to examine whether the duration of breastfeeding during infancy was associated with the time of spontaneous resolution of monosymptomatic enuresis (SRME).

**Materials and Methods:**

A total of 1500 people were surveyed at four centers. One hundred and eighty-one people with a history of monosymptomatic enuresis (ME) who received no treatment and had no day time symptoms were included in the study. The relationship between the duration of breastfeeding and SRME was assessed by considering the duration of breastfeeding as both continuous and categorical (cut-off value 5 months) variable. The multivariate general linear model was used to identify independent predictors such as gender, family history, and educational status of parents.

**Results:**

Pearson correlation analysis of the age of SRME and duration of breastfeeding found no statistically significant relationship. However, there was a significant difference in the age of SRME of those who were breastfed for 5 months or less compared to those who were breastfed for more than 5 months. According to the multivariate analysis, gender and educational status of parents were not effective on the age of SRME. Stepwise linear regression model showed that breastfeeding for five months or less and family history could affect the age of SRME. The regression formula was: age of SRME=9.599 + (3.807×five months or less of breastfeeding) + (1.258×positive family history).

**Conclusions:**

It was found that when breastfeeding lasted for more than 5 months, there was a positive contribution to SRME.

## INTRODUCTION

Enuresis is an important and frequently seen health problem during childhood throughout the world and it is predicted that there are over 50 million children with enuresis worldwide (1). According to the definition of the International Children’s Continence Society, in the absence of attendant symptoms of the lower urinary tract, such as daytime urinary frequency, urgency, hesitancy, straining, daytime incontinence etc., any wetting during sleep above the age of 5 years is defined as monosymptomatic enuresis (ME) (1-3). The overall prevalence of ME declines by about 15% each year with increasing age, occurring in 15% of children aged 5 years, and 7% aged 7 (2, 4). Although ME is less commonly seen after the age of 7 years, 1-2% of adults are still enuretic (3, 5, 6).

ME has been accepted as a common bio-behavioral problem in early childhood (4). The etiology seems to be multifactorial and several etiologies have been asserted for ME, including developmental delay, immature bladder function, immature sleep pattern and insufficient nocturnal antidiuretic hormone (7, 8). Most of these etiologies for ME are related to delayed development because they are normally seen in younger children and infants. The developmental theory is supported by clinical studies that report more developmental delays in children with ME compared with controls (8, 9). Other causes of ME include psychosocial and familial factors. The effects of psychosocial factors such as stress are ambiguous but have been stated to be associated with ME (7, 10, 11). Also, genetic factors have been recognized in ME and familial clustering has been observed in several studies (7-9, 12).

Although the high rate of spontaneous resolution is obvious, there are no articles investigating the effect of the factors that contribute to the time of spontaneous resolution of monosymptomatic enuresis (SRME). We examined the relationship between ME and breastfeeding because both have been reported to be strongly associated with childhood development. For example, there is a lot of clinical evidence that breastfeeding may provide neurodevelopmental advantages to children (13-16). Neurodevelopmental delays have been identified in children with ME (9, 12). In addition to this, it was determined that psychogenic factors similar to stress play a role in the etiology of ME, but breastfeeding has been shown to have positive contributions to self-esteem and some psychological diseases (17). According to numerous studies, it is clear that both breastfeeding and ME are associated with child development, so the objective of this study was to examine whether duration of breastfeeding during infancy was associated with the time of SRME.

## MATERIALS AND METHODS

### Patient Population

After receiving permission from the institutional ethics committee, about 1500 people were surveyed. The questionnaires were obtained by talking to individuals face to face in four referral hospitals which were located in different cities in Turkey. Only patient relatives, not the patients, were asked to answer the questionnaire in different out-patient clinics of the centers (outpatient clinics of urology, dermatology and family medicine). If the answer to the question of whether there was bed-wetting after the age of five (the 60th month after birth) was yes, the other questions were asked. To evaluate the spontaneous resolution correctly, patients who were given any treatment for bed-wetting were excluded. In those whose bed-wetting resolved spontaneously, the duration of breastfeeding, educational status of parents and family history of enuresis were questioned. The presence of ME in first degree relative was evaluated as positive family history. We excluded patients with diurnal enuresis or with any day-time symptoms such as pollakiuria, frequency, and urgency during any part of their life. Patients with illnesses which could affect micturition habits such as diabetes mellitus or neurological diseases were also excluded. Because the basis of the study is information about infancy, those over the age of 35 were excluded from the study. If the participating children were aged 16 and below, information provided by the parents of children were taken into consideration. The information given by participants who were older than 16 was confirmed by another member of the family. At the end of the questionnaire, patients and parents were all asked if they were extremely sure about the information they provided. If the answer was no, they were excluded from the study. If the double checks were inconsistent, participants were also excluded from the study. As a result, the clinical data from 181 people were evaluated.

### Statistical analysis

All statistical analyses were performed using SPSS, version 20.0. All values are shown as mean ± standard deviation. The normal distribution of the sample data was checked with the Kolmogorov-Smirnov and Shapiro-Wilk tests (18). The comparison of duration of breastfeeding and age of resolution of ME was completed using the Pearson correlation analysis. The effect on SRME of gender, family history and five months and less or longer duration of breastfeeding were evaluated with the Mann-Whitney U test. The Kruskal-Wallis tests were conducted to compare the age of SRME and the educational status of mother and father (each had 3 subgroups). The multivariate general linear model and stepwise linear regression were used to identify independent predictors such as gender, family history, educational status of parents, and five months or less duration of breastfeeding. The model fit was assessed using appropriate residual and goodness-of-fit statistics. A p value of <0.05 was accepted as significant.

## RESULTS

The study consisted of 103 male patients (56.9%) and 78 female patients (43.1%); 181 patients in total. The average age was 25.7±6.2 (between 7-35 years), 90% were over the age of 14. The average age of SRME was 11.0±3.5 years (range 5-30 years) and the average duration of breastfeeding was 16.5±9.8 months (range 0-60 months) (Table-1).

**Table 1:** The demographic characteristics of patients.

	n	Mean ± ss	Median	Minimum	Maximum
**Age**	181	25.7 ± 6.2	27.0	7.0	35.0
**Age of spontan resolution of MNE**	181	11.0 ± 3.5	11.0	5.0	30.0
**Duration of breatfeeding (month)**	181	16.5 ± 9.8	13.0	0.0	60.0

**Figure 1:**
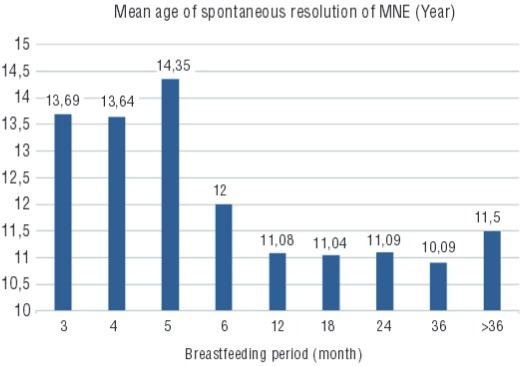
The relationship between breastfeeding and mean age of spontaneous resolution of monosymptomatic enuresis is demonstrated.

Pearson correlation analysis of the age of SRME and duration of breastfeeding found no statistically significant relationship (p=0.250) (Figure-1). However, there was a significant difference in the age of SRME of those who were breastfed for 5 months or less compared to those who were breastfed for more than 5 months (average age of resolution for those breastfed ≤5 months 14.35±5.0 years; for those breastfed >5 months 10.58±2.9; p<0.001). There were no statistically significant relationships between the age of SRME and gender or presence of family history (Table-2). There was no significant correlation found between the age of SRME and educational status of parents (Table-3). A multivariate linear model was used to evaluate the independent predictors for the age of SRME. According to this analysis, the age of SRME was not affected by gender, or the educational status of mother and father (p=0.483, p=0.488, p=0.396, respectively). Stepwise linear regression showed that breastfeeding for five months or less and family history could affect the age of SRME (Table-4). The regression formula was: age of SRME=9.599 + (3.807×five months or less of breastfeeding) + (1.258×positive family history).

**Table 2:** Gender, breastfeeding, family history and SRMNE.

		Age of spontaneous resolution					Mann-Whitney U Test		
		
		n	Mean ± ss	Med	Min	Max	Mean Rank	U	p
**Gender**	Female	78	10.99 ± 3.80	10.0	6.0	30.0	88.00	3783	0.501
	Male	103	11.01 ± 3.23	11.0	5.0	18.0	93.27		
	All	181	11.00 ± 3.48	11.0	5.0	30.0			
**Duration of breastfeeding**	≤ 5 month	20	14.35 ± 5.08	13.0	8.0	30.0	129.63	837.5	0.0001
	> 5 month	161	10.58 ± 2.99	10.0	5.0	22.0	86.20		
	All	181	11.00 ± 3.48	11.0	5.0	30.0			
**MNE in family history**	Apsent	40	10.08 ± 3.16	10.0	5.0	17.0	77.80	2292	0.07
	Existent	141	11.26 ± 3.53	11.0	5.0	30.0	94.74		
	All	181	11.00 ± 3.48	11.0	5.0	30.0			

**Table 3:** Education status of parents and SRMNE.

		Age of spontaneous resolution					Kruskal-Wallis H Test		
		
		n	Mean ± ss	Med	Min	Max	Mean Rank	H	p
Education status of mother	Primary school and less	122	10.97 ± 3.28	11.0	5.0	22.0	89.82	0.05	0.975
	High school	34	11.26 ± 4.43	11.0	6.0	30.0	89.69		
	University	25	10.80 ± 3.07	10.0	5.0	17.0	91.61		
	Total	181	11.00 ± 3.48	11.0	5.0	30.0			
Education status of father	Primary school and less	110	10.91 ± 3.00	11.0	5.0	21.0	91.77		
	High school	46	11.24 ± 4.61	10.0	6.0	30.0	88.23		
	University	25	10.96 ± 3.13	11.0	5.0	18.0	92.70		
	Total	181	11.00 ± 3.48	11.0	5.0	30.0		0.18	0.914

**Table 4:** Results of the multivariate general linear model analysis.

	B	Beta	p	CI 95%
**Constant**	9.599		0.0001	8.568-10.630
**Duration of breatfeeding (0: five month or less 1: longer than five month)**	3.807	0.344	0.0001	2.288-5.327
**Family history (0: absent – 1 present)**	1.258	0.151	0.032	0.110-2.406

## DISCUSSION

This is the first study to investigate the relationship between the age of SRME in children and breastfeeding. ME is regarded as a self-limiting condition and expected to resolve spontaneously at a rate of 15% per year (5, 19, 20). Alarm therapy and desmopressin, which are evidence-based first-line treatments, are recommended for current treatment of ME (3, 21). Some parents who have children with ME choose an observational approach instead of the treatment option. However, in spite of this high rate of spontaneous resolution, there is no study researching the factors that could affect this rate found in the literature.

There may be various factors, such as severity of symptoms, which influence the age of spontaneous resolution. Although many factors such as behavior abnormalities, immature bladder function, imamture sleep pattern, insufficient nocturnal antidiuretic hormone and hereditary factors can be coupled with ME, there is a traditional opinion that developmental immaturity of voiding control is the main reason (7, 8). The role of stress and psychological factors in the pathogenesis of ME has been shown in a variety of studies (7, 10, 11, 21). Recently many studies have identified a delay in neuromotor development in children with ME (8, 9, 12, 22). These studies assert a maturational deficit of the brainstem as the possible central dysfunction of the disorder.

It is known that breastfeeding has a positive effect on behavior problems. Breastfeeding forms a bond between mother and baby and a variety of studies have shown many positive psychological effects like increasing self-esteem and preventing depression (17, 23, 24). According to a study by Kwok et al. durations of breastfeeding of less than 3 months were linked to worse behavior and lower self-esteem (17). Additionally, a range of studies have investigated the effects of breastfeeding on cognitive development and neurodevelopment (13, 15, 16, 25). The central nervous system has the second highest concentration of lipids in the body after adipose tissue, which contains predominantly triglycerides. The lipids in the brain are present as the structural phospholipid components of cell membranes (25). Docosahexaenoic acid and arachidonic acid, which are present in breast milk, are examples of the most important and basic long chain polyunsaturated fatty acids. Babies only have a limited capability to synthesize such fats from precursors (14). It has been shown that especially the higher n-3 and n-6 long-chain polyunsaturated fatty acids in breast milk have important effects on neural and visual development (14, 16, 25). Vestergaard et al. determined that the duration of breastfeeding was a specific milestone for motor skills and early language development (16).

In the literature there were few studies investigating the relationship between breastfeeding and ME; however, none investigated the relationship between spontaneous resolution and breastfeeding. In a cross-sectional study by Gumus et al., different clinical factors in childhood bed-wetting were evaluated and, taking rates of breastfeeding in the first four months after birth as reference, there was no difference found between cases with enuresis and those without (26). Singh et al. investigated the relationship between bed-wetting and several different clinical features of 100 children. Although there was no control group in the study, the authors found higher enuresis prevalence in children fed using a bottle compared with those who were breastfeed (27).

A study by Barone et al. compared enuretic children with a control group and showed that duration of breastfeeding longer than 3 months had a protective effect against bed-wetting (28). In a recent observational case-control study involving 200 children and adolescents from 6 to 14 years old, Oliviera et al. indicated that the duration of breastfeeding of less than 4 months is strongly associated with primary enuresis (29). In our study while there was no significant difference found between the age of spontaneous resolution and breastfeeding of children with ME, comparing the group who were breastfed for 5 months or less with those fed for longer, breastfeeding had a significant positive contribution to age of SRME (p<0.001). With regard to multivariate analysis, gender and educational status of mother and father were not effective on the age of SRME. Stepwise linear regression model showed that five months or less of breastfeeding and family history could affect the age of SRME. There was very weak correlation between the family history and the age of SRME. Accordingly, breastfeeding for at least 5 months can be expected to provide a contribution to the age of SRME in children. This conclusion correlates with the results of previous studies and is in accordance with our expectations due to the positive contribution on neurodevelopment and childhood psychology of breastfeeding.

None of the patients included in the study after the survey had treatment of ME in their history. However, due to the psychological effects of bed-wetting on the child and family, especially in school-going children, treatment is recommended (30). As a result, when designing prospective studies about SRME in children, it may not be ethical not to give treatment. As a result, a survey questionnaire was used for the study design. It may be considered that a case control approach would have been more appropriate. However, we investigated the factors affecting the time of SRME and it is impossible to evaluate the time of SRME in patients who were not bedwetting which is why we did not design a case control study.

The major limitation of the current study was the long time between breastfeeding, enuresis and application of the questionnaire. Nevertheless, in order to increase the reliability of data, those over the age of 35 were excluded from the study, information given only by the family in children under the age of 16 were taken into consideration, and information given by participants over the age of 16 were double-checked. Another limitation of the study was that enuresis-related diseases such as adenoid hyperplasia and obstructive sleep apnea-hypopnea syndrome were not considered in the study design.

## CONCLUSIONS

One of the frequently observed disorders in childhood is ME, generally evaluated as a benign situation due to the high rate of spontaneous resolution. If it continues into the school-going period, treatment is recommended due to negative effects on the child and family, but this topic is still debated. In our study lengthening the duration of breastfeeding to more than 5 months was found to have an effect on spontaneous resolution. Before planning medication or other treatments for children with ME, the factors that can affect spontaneous resolution should be evaluated to reduce the cost of medications or other treatments and to protect children from the side effects of medication treatment. Breastfeeding, with no costs, has a positive effect on the age of SRME, similar to its effects on many aspects of childhood development. This under-researched topic requires more prospective randomized studies.
